# Modulation of Comorbid Chronic Neuropathic Pain and Anxiety-Like Behaviors by Glutamatergic Neurons in the Ventrolateral Periaqueductal Gray and the Analgesic and Anxiolytic Effects of Electroacupuncture

**DOI:** 10.1523/ENEURO.0454-23.2024

**Published:** 2024-08-23

**Authors:** Xixiao Zhu, Chi Zhang, Yuxin Hu, Yifang Wang, Siqi Xiao, Yichen Zhu, Haiju Sun, Jing Sun, Chi Xu, Yunyun Xu, Yuerong Chen, Xiaofen He, Boyu Liu, Jinggen Liu, Junying Du, Yi Liang, Boyi Liu, Xiaoyu Li, Yongliang Jiang, Zui Shen, Xiaomei Shao, Jianqiao Fang

**Affiliations:** Key Laboratory of Acupuncture and Neurology of Zhejiang Province, Department of Neurobiology and Acupuncture Research, The Third Clinical Medical College, Zhejiang Chinese Medical University, Hangzhou 310053, China

**Keywords:** anxiety-like behaviors, CamkIIα, chronic neuropathic pain, electroacupuncture, ventrolateral periaqueductal gray

## Abstract

Comorbid chronic neuropathic pain and anxiety is a common disease that represents a major clinical challenge. The underlying mechanisms of chronic neuropathic pain and anxiety are not entirely understood, which limits the exploration of effective treatment methods. Glutamatergic neurons in the ventrolateral periaqueductal gray (vlPAG) have been implicated in regulating pain, but the potential roles of the vlPAG in neuropathic pain-induced anxiety have not been investigated. Herein, whole-cell recording and immunofluorescence showed that the excitability of CamkIIα neurons in the vlPAG (vlPAG^CamkIIα+^ neurons) was decreased in mice with spared nerve injury (SNI), while electroacupuncture (EA) activated these neurons. We also showed that chemogenetic inhibition of vlPAG^CamkIIα+^ neurons resulted in allodynia and anxiety-like behaviors in naive mice. Furthermore, chemogenetic activation of vlPAG^CamkIIα+^ neurons reduced anxiety-like behaviors and allodynia in mice with SNI, and EA had a similar effect in alleviating these symptoms. Nevertheless, EA combined with chemogenetic activation failed to further relieve allodynia and anxiety-like behaviors. Artificial inhibition of vlPAG^CamkIIα+^ neurons abolished the analgesic and anxiolytic effects of EA. Overall, our study reveals a novel mechanism of neuropathic pain-induced anxiety and shows that EA may relieve comorbid chronic neuropathic pain and anxiety by activating vlPAG^CamkIIα+^ neurons.

## Significance Statement

Neuropathic pain is clinically accompanied by anxiety. Both glutamatergic neurons in the ventrolateral periaqueductal gray (vlPAG) and electroacupuncture (EA) have demonstrated analgesic properties. However, the efficacy of these interventions in addressing neuropathic pain and its concomitant anxiety has yet to be fully elucidated. Chemogenetic activation of vlPAG^CamkIIα+^ neurons not only resulted in analgesia but also mitigated anxiety-like behaviors in SNI mice, mirroring the effects observed with EA treatment. Conversely, inhibition of vlPAG^CamkIIα+^ neuron activity in naive mice reduced pain thresholds and induced anxiety-like behavior, while also negating the beneficial effects of EA. These findings provide novel insights into the mechanistic interplay between chronic neuropathic pain and anxiety, highlighting the therapeutic potential of targeting vlPAG glutamatergic neurons in these conditions.

## Introduction

Chronic neuropathic pain is pain caused by diseases or lesions of the somatosensory nervous system, and ∼6.9–10% of adults suffer from neuropathic pain (van Hecke et al., 2014). Chronic pain is commonly accompanied by psychological conditions, including anxiety and depression (Feingold et al., 2018; Michaelides and Zis, 2019). Epidemiological studies have reported that ∼45% of patients with chronic pain screen positive for anxiety disorders (Asmundson and Katz, 2009; Battaglia et al., 2020). An increasing number of studies have reported that chronic pain-induced anxiety leads to reduced immune function, impaired decision-making, and worsened insomnia (Palmer and Alfano, 2020). Analgesic drugs, antidepressants, and antianxiolytic agents can somewhat alleviate comorbid pain and anxiety, but these drugs are associated with a high recurrence rate and side effects (Feighner, 1999). These include gastrointestinal adverse reactions, drug addiction, and hypertension (Bair et al., 2004). Electroacupuncture (EA) can reduce chronic neuropathic pain (Lee et al., 2014; He et al., 2022), insomnia, and anxiety (Yin et al., 2022) in clinical settings. Hence, EA may be a potential treatment option for comorbid chronic pain and anxiety with few side effects. As comorbid pain and anxiety is thought to be associated with central nervous system dysfunction (Hassett et al., 2014), it is necessary to identify the key brain regions and neurons involved in this disease. However, at present, the underlying mechanism of comorbid chronic pain and anxiety is still unclear (Johannes et al., 2010).

Recent studies have shown that the brain regions associated with pain and anxiety partly overlap and include the rostral anterior cingulate cortex (rACC), ventrolateral periaqueductal gray (vlPAG), dorsal raphe nucleus (DRN), and amygdala (Bushnell et al., 2013; Samineni et al., 2017; Liang et al., 2020). Previously, we found that activation of the rACC^Glu^→vlPAG circuit could regulate chronic pain-induced anxiety (X. Zhu et al., 2021). Notably, the vlPAG is the key brain region of the emotion and descending pain modulatory systems and mainly contains glutamatergic neurons, GABAergic neurons, and serotonergic neurons (Lü et al., 2010; Pati and Kash, 2021; Yu et al., 2021). However, the neuronal type in the vlPAG that is involved in the regulation of chronic pain-induced anxiety has not been determined. Several researchers have noted that the excitability of GABAergic neurons in the vlPAG is increased and that the excitability of glutamatergic neurons is inhibited in mice with chronic pain (H. Zhu et al., 2019). Moreover, psychological stress-induced negative emotional behavior is related to a decrease in glutamate transmission in the vlPAG (Peng et al., 2022). In mice with inflammatory pain, glutamatergic transmission in the vlPAG mediates stress-induced negative emotional behaviors (Ko et al., 2020), and enhancement of glutamatergic transmission rescues these behaviors (Chou et al., 2018). Other researchers have shown that changes in glutamatergic neurons, which represent many of the neurons in the vlPAG, can affect the paw withdrawal mechanical threshold (Albrecht et al., 2010; Tovote et al., 2016). However, whether glutamatergic neurons in the vlPAG are involved in the regulation of chronic neuropathic pain-induced anxiety has not been determined.

As an effective analgesic therapy, EA has been widely used clinically. Both clinical and experimental studies have shown that EA can effectively relieve allodynia (Zhao et al., 2019a; X. Yang et al., 2020; Kim et al., 2021) and reduce drug tolerance when combined with antidepressants (Zhao et al., 2019b). Clinical research studies have identified EA as an effective treatment for anxiety disorders and other negative emotions without significant adverse effects (Amorim et al., 2018; M. Wu et al., 2022; F. Zhou et al., 2022; Yin et al., 2022). Several basic studies have reported that EA can relieve allodynia by regulating glutamate transporters in pain model rats (Wang et al., 2022). W. Zhou et al. (2007) assessed the relationship between EA-mediated opioidergic modulation of visceral cardiovascular responses and glutamate and found that EA can regulate glutamate. In our previous experiments, we demonstrated that EA can exert analgesic effects through the rACC^Glu^→vlPAG (X. Zhu et al., 2021) pathway. Although EA has been shown to be effective at relieving allodynia and emotional disorders (Sun et al., 2013; Kondo and Kawamoto, 2014), the mechanism underlying the analgesic and anxiolytic effects of EA needs to be further studied. Importantly, whether EA exerts analgesic and anxiolytic effects through vlPAG glutamatergic neurons is still unclear.

In this study, we combined electrophysiological recording, immunofluorescence, and chemogenetics to investigate the changes in the excitability of vlPAG CamkIIα^+^ neurons in the context of chronic neuropathic pain-induced anxiety. We subsequently aimed to explore whether EA alleviates allodynia and anxiety-like behaviors through vlPAG CamkIIα^+^ neurons.

## Materials and Methods

### Animals

All procedures were approved by the guidelines of the Laboratory Animal Management and Welfare Ethical Review Committee (Permission Number: IACUC-20190225-01). Wild-type male C57BL/6J mice (aged 8–12 weeks) were subjected to virus injection and behavioral experiments. Male mice (aged 4–6 weeks) were subjected to in vitro electrophysiology. The mice were housed in groups of five in plastic cages containing corn cob bedding. Adaptive feeding was carried out in a laboratory for 7 d prior to the experiment. The ambient environment was kept stable (room temperature of 23–25°C and humidity of 50–60%), and plenty of water and fodder were provided. The animals were maintained on a 12 h light/dark cycle (lights on from 7:00 to 19:00).

### SNI model construction

**Figure 1.**
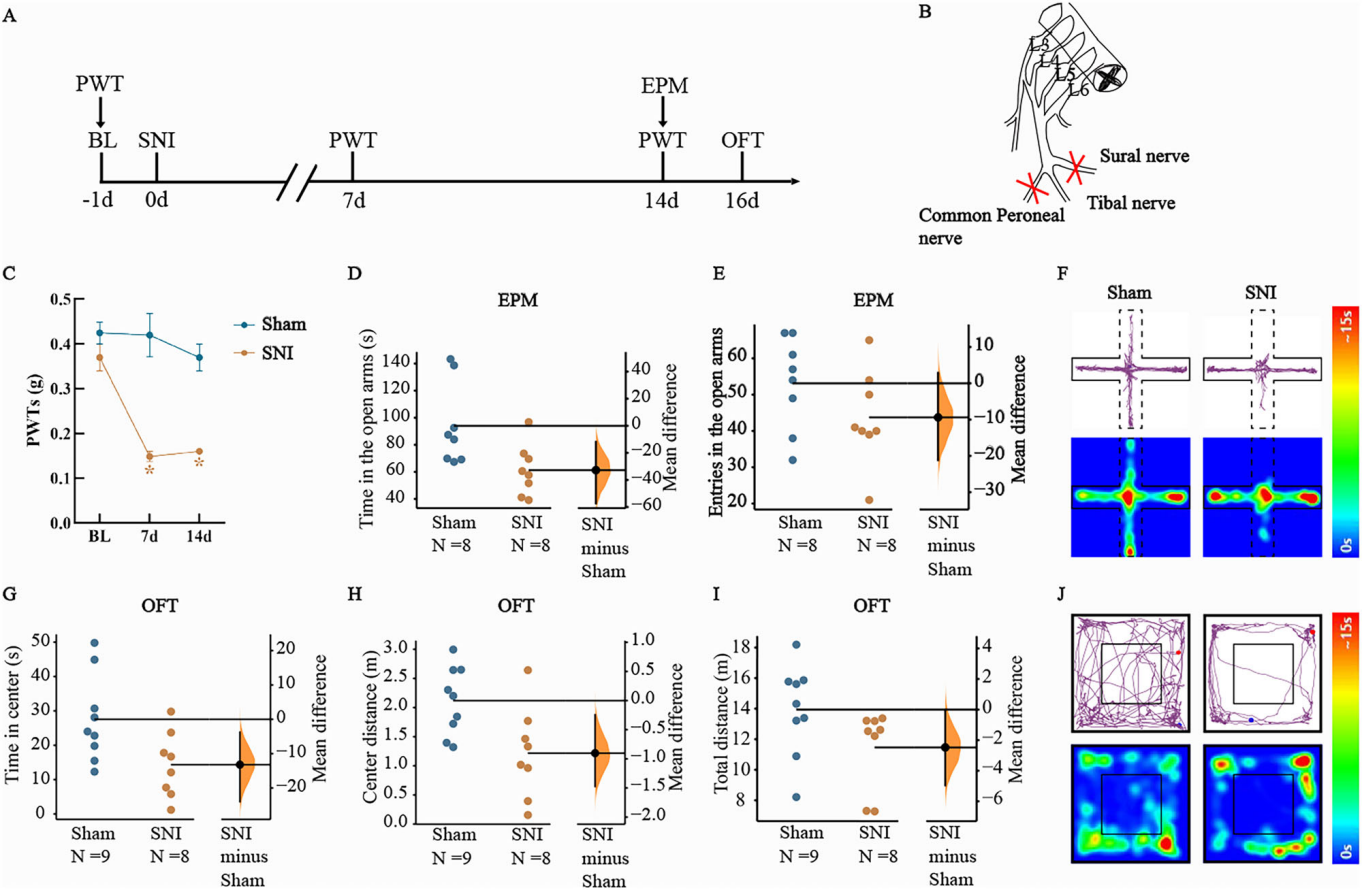
SNI induces allodynia and anxiety-like behaviors. ***A***, Schematic diagram of the experimental process. ***B***, Schematic of the SNI surgical procedure. ***C***, Mechanical PWTs of the sham group (blue) and SNI group (red) after SNI surgery. *N* = 8 per group. The data are presented as the means ± SEMs. *p < 0.05 versus the sham group. ***D***, ***E***, Quantitative analysis of the time spent in the open arms and number of entries into the open arms in the EPM test in the sham and SNI groups. *N* = 8 per group. ***F***, Representative diagrams showing the movement trajectories and activity heatmaps of the two groups in the EPM. ***G–I***, Quantitative analysis of the time spent in the center, distance traveled in the center and total distance traveled in the OFT in the sham and SNI groups. *N* = 8–9 per group. ***J***, Representative diagrams showing the movement trajectories and activity heatmaps of the two groups in the OFT. The mean difference between the sham and SNI groups is shown in the above Cumming estimation plots. The raw data are plotted on the left axis; the mean difference is plotted on the floating axis on the right as a bootstrap sampling distribution. The mean differences are shown as dots; the 95% confidence intervals are shown by the vertical error bars.

Surgery was performed as previously described (Peirs et al., 2021). Briefly, the mice were deeply anesthetized via inhalation of 2.5% isoflurane via a mobile respiratory anesthetic machine. In the SNI group, the hair on the left hindlimb was removed with scissors, and the skin was exposed. After disinfection with iodophor and ethanol, a small incision was made at the midpoint between the greater trochanter of the femur and the knee, and the subcutaneous muscles were separated by blunt dissection to expose the three branches of the sciatic nerve, including the sural, common peroneal, and tibial nerves. The common peroneal and sural nerve branches were tightly ligated with 6-0 nylon sutures and transected below the ligature (Fig. 1*B*), and 2–3 mm of the nerve was removed distal to the ligature. The tibial nerve was kept intact. The muscle tissue and skin were sutured layer by layer with 6-0 nylon sutures (Peirs et al., 2021). The sham group underwent the same procedure, but the nerves were kept intact.

### EA treatment

The therapeutic effect of EA on SNI in mice and the underlying mechanism were investigated in this study. EA treatment was administered at specific time points (8, 10, 12, 14, and 16 d) following SNI. The bilateral Zusanli (ST36) and Sanyinjiao (SP6) acupoints were selected for EA treatment according to previous research (Y. Y. Wu et al., 2015). During the treatment, all the mice were immobilized. Then, 0.16 × 7-mm-long acupuncture needles were inserted into the acupoints, and the end of each needle was connected to a HANS acupuncture point nerve stimulator (HANS-200A). The stimulation parameters used were as follows: a frequency of 2 Hz, a duration of 30 min, and an intensity of 0.3 mA. In the sham EA group, 0.16 × 7 mm acupuncture needles were inserted into the same acupoints and connected to the HANS-200A stimulator, but no electrical stimulation was applied. The other group of mice was immobilized in the same way but did not receive EA treatment. EA treatment was administered 30 min after the mice were injected intraperitoneally with clozapine *N*-oxide (CNO).

### Von Frey filament test

The PWT of the mice was investigated with von Frey filaments in this study. All the mice were placed in an opaque Plexiglas cage on a grid platform for 1 h before testing, after which the PWT of the left hindpaw was determined. Starting with the 0.02 g filament, each filament was applied to the left hindpaw, and the force was gradually increased. Paw withdrawal or shaking or licking of the paw was considered to indicate positive responses. The filaments were applied at intervals of >1 min. The response to each stimulus was recorded, and the force at which positive responses occurred three out of five times was considered the PWT. The mechanical pain threshold was measured at −1, 7, and 14 d after SNI. Adaptive pain was assessed 2 d before the baseline pain threshold was measured.

### Assessment of anxiety-like behaviors

The behavioral tests were performed in a dimly lit (∼20 lux) room. The mice were then transported to the behavioral testing room for habituation at least 1 d before testing. The trial conditions were as follows: temperature, 23–25°C; humidity, 50–60%; and noise level <40 dB. The behavioral tests were started 30 min after injection of CNO or 0.9% saline. All the behavior tests were videotaped using a video tracking system.

### Elevated plus maze test

To explore the changes in anxiety-like behavior in mice, motor activity in an elevated plus maze (EPM) was assessed at 14 d after SNI. The EPM was elevated 35 cm above the ground and consisted of two closed arms (30 × 6 × 15 cm), two open arms (30 × 6 cm), and a central area (6 × 6 cm). At the beginning of the test, a mouse was placed in the open arm with its head facing the central area. After 30 s of free exploration and adaptation, the mouse was allowed to explore the maze for 5 min, and the activity of the mouse was recorded with the ANY-maze video tracking system. The maze was wiped with 75% alcohol and double-distilled water between each test.

### Open field test

To explore changes in the anxiety-like behavior of the mice, the open field test (OFT) was performed in an uncovered wooden box (40 × 40 × 40 cm) 16 d after SNI. The bottom surface was divided into 16 square grids, with the middle four grids defined as the central zone and the other 12 grids defined as the peripheral zone. At the beginning of the experiment, a mouse was placed in the central area and allowed to freely explore the entire box for 5 min. The maze was wiped with 75% alcohol and double-distilled water between each test.

### Stereotactic injections

For chemogenetic virus injections, the mice were anesthetized with 0.3% sodium pentobarbital (60 mg/kg, i.p.) and mounted in a stereotaxic frame (RWD, 68025). Injections into the vlPAG [anteroposterior (AP): −4.7 mm; lateral (ML): ±0.5 mm; ventral (V): −2.14 mm] were performed using a glass microelectrode connected to an infusion pump (WPI, UMC4) at a rate of 60 nl/min. A volume of 80 nl of the virus was injected into the vlPAG.

To determine the effect of chemogenetic regulation of vlPAG neurons on behavior, AAV2/9-CaMKIIα-hM3Dq-mCherry (3.04 × 10^12^ vg/mg; BrainVTA) was injected into the right vlPAG. AAV2/9-CaMKIIα-hM4Di-mCherry (3.38 × 10^12^ vg/ml; BrainVTA) was injected into the bilateral vlPAG. CNO (1 mg/ml; BrainVTA) was injected (2 mg/kg, i.p.) into the mice at 8, 10, 12, 14, and 16 d after SNI. Images of virus expression were obtained using a virtual slide microscope (VS120-S6-W; Olympus).

### Whole-cell recording

For electrophysiological recording of vlPAG neurons, AAV2/9-CaMKIIα-mCherry (3.04 × 10^12^ vg/mg; BrainVTA) was injected into the right vlPAG (AP: −4.7 mm; ML: ±0.5 mm; DV: −2.14 mm). The mice were anesthetized with 0.3% pentobarbital sodium and placed in a stereotaxic framework. A volume of 80 nl of virus was injected into the vlPAG by glass microelectrodes connected to infusion pumps at a rate of 60 nl/min. At least 21 d after virus injection, the mice were anesthetized with avertin (240 mg/kg, i.p.). The mice were decapitated, and their brains were quickly removed and placed in an ice-cold solution containing the following (in mM): 234 sucrose, 5 KCl, 1.25 NaH_2_PO_4_, 5 MgSO_4_, 26 NaHCO_3_, 25 dextrose, and 1 CaCl_2_, pH 7.2–7.4 (oxygenated with 95% O_2_ and 5% CO_2_). Coronal brain slices (300 μm) containing the vlPAG were cut with a vibratome (VT-1200S, Leica). The slices were subsequently placed in artificial cerebrospinal fluid (ACSF) supplemented with the following (in mM): 125 NaCl, 3 KCl, 1.25 NaH_2_PO_4_, 1 MgCl_2_, 26 NaHCO_3_, 25 dextrose, and 2 CaCl_2_, pH 7.2–7.4 (oxygenated with 95% O_2_ and 5% CO_2_) for 1 h and subsequently maintained at 25°C for further experiments. The slices were transferred to the recording chamber of an FN1 microscope (Nikon) and visualized with an IR CCD camera (DAGE-MTI). For current-clamp recordings, the slices were transferred to a 25°C recording chamber perfused with 2–3 ml/min ACSF (the same composition as above). Patch pipettes were pulled from glass microelectrodes at resistances of 6–9 MΩ and filled with the following (in mM): 135 KCH_3_SO_3_, 4 KCl, 2 NaCl, 10 HEPES, 4 Mg-ATP, 0.3 Tris-GTP, and 7 Tris2-phosphocreatine. Patch-clamp recordings were performed by a MultiClamp 700B patch-clamp amplifier (Axon). The series resistance was 10–20 MΩ. For current injection, pyramidal neurons expressing the AAV2/9-CaMKIIα-mCherry virus in the right vlPAG were current clamped, and −200 to 220 pA hyper- and depolarizing currents with a 10 pA step were injected for 500 ms.

### Immunohistochemistry

Immunohistochemistry was applied in this study. The mice were deeply anesthetized with 0.3% pentobarbital sodium and transcranially perfused with 0.9% saline followed by 4% paraformaldehyde. Notably, for the c-Fos staining experiments, the animals were killed 1.5 h after behavior testing. The brains were removed, stored in 4% paraformaldehyde at 4°C, and then dehydrated in 15 and 30% sucrose until they sank. Coronal sections (20 µm) were cut on a cryostat freezing microtome (Thermo Fisher Scientific, NX50). For immunofluorescence, the brain sections were rewarmed at 37°C for 1 h and then washed with TBST six times for 8 min. The sections were then incubated with 10% donkey serum for blocking at 37°C for 1 h. The primary antibodies used were as follows: mouse monoclonal anti-vGLUT2 (1:100; Sigma, MAB5504), rabbit monoclonal anti-c-Fos (1:800; rabbit, Abcam, ab190289), rat polyclonal anti-c-Fos (1:800; sysy226004; Synaptic Systems), and rabbit polyclonal anti-glutamate (1:800; catalog #G6642; Sigma). The brain sections were incubated with primary antibodies overnight at 4°C and rinsed with TBST. Then, the sections were incubated with Alexa 488- or Alexa 647-conjugated secondary antibodies at 37°C for 1 h. The brain sections were then washed and mounted on slides in media containing DAPI (ab104139; Abcam). Fluorescence images were captured using a virtual slide microscope (VS120-S6-W; Olympus).

### Statistical analysis

The data are expressed as the mean ± standard error of the mean (SEM). The data were analyzed with SPSS (version 17.0; IBM) and Prism 8 (GraphPad Software). Wherever possible, we used Cumming estimation plots to determine effect sizes and confidence intervals (J. Ho et al., 2019). One-way ANOVA or two-way ANOVA followed by Tukey's post hoc test was used for multiple comparisons. An unpaired *t* test was used for comparisons between two groups. All the PWT and firing rate data were analyzed by two-way ANOVA followed by Tukey's post hoc test. The EPM and OFT data were analyzed by one-way ANOVA followed by Tukey's post hoc test. Independent-sample *t* tests were performed to evaluate c-Fos expression. The significance of the differences is reported in the figure legends. A *p* < 0.05 was considered to indicate statistical significance for all analyses.

## Results

### Chronic neuropathic pain model mice exhibit allodynia and anxiety-like behaviors

A mouse model of chronic neuropathic pain-induced anxiety was established using spared nerve injury (SNI; the experimental timeline is shown in Fig. 1*A*). The tibial nerve was preserved, while the sural and common peroneal nerves were cut (Fig. 1*B*). We used von Frey filaments to assess the mechanical paw withdrawal threshold (PWT) of the left hindpaw. Seven days and 14 d after SNI, the PWT of the SNI group was significantly lower than that of the sham group (two-way RM ANOVA: *F*_(2,42) _= 12.41, *p *< 0.001; Tukey's post hoc test: *p *< 0.05, *p *< 0.05; Fig. 1*C*). The results showed that the SNI model was successfully established and that SNI-induced mechanical allodynia lasted at least 14 d. The anxiety-like behaviors of the mice in all the groups were assessed at 14 and 16 d after SNI using the EPM test and OFT, which are commonly used to measure anxiety-related behaviors (Carola et al., 2002; Fernandes et al., 2021). We found that SNI model mice spent less time in the open arms of the EPM than did sham group mice (*t* = 2.591; mean difference = −32.762 [95.0% CI −57.18, −11.937]; *p *< 0.05; Fig. 1*D*). There was no significant difference in the number of entries into the open arms of the EPM between the two groups (*t* = 1.455; mean difference = −9.375 [95.0% CI −21.13, 2.875]; *p *> 0.05; Fig. 1*E*). The SNI group spent less time and traveled a shorter distance in the center of the open field than the sham group did (*t* = 2.401; mean difference = −13.19 [95.0% CI −23.94, −3.779]; *p *< 0.05; Fig. 1*G*; *t* = 2.703; mean difference = −0.90 [95.0% CI −1.47, −0.244]; *p *< 0.05; Fig. 1*H*). The total distance traveled in the OFT was not significantly different between the two groups (*t* = 2.474; mean difference = −2.47 [95.0% CI −4.95, −0.037]; *p *> 0.05; Fig. 1*I*). Representative diagrams showing the movement trajectories and activity heatmaps of the two groups in the EPM and OFT are shown in Figure 1*F*,*J*, respectively. These results indicate that SNI could induce nociceptive sensitization accompanied by anxiety-like behaviors in mice.

### The excitability of vlPAG^CaMKIIα+^ neurons in SNI model mice was decreased

**Figure 2.**
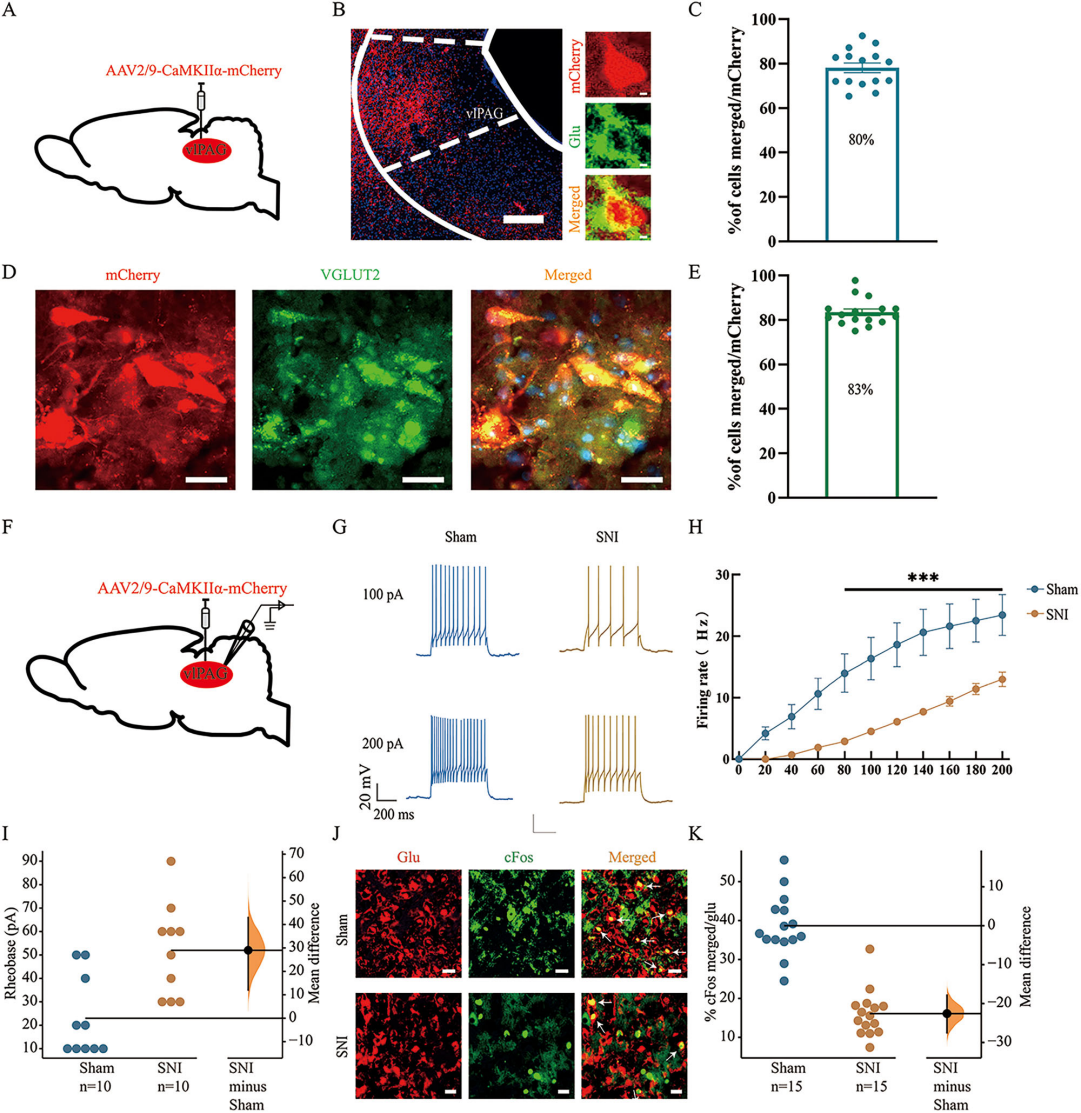
The excitability of vlPAG^CaMKIIα+^ neurons in SNI model mice was decreased. ***A***, Schematic diagram of chemogenetic virus injection into C57BL/6 mice in the sham group. ***B***, Representative immunofluorescence image showing the localization of glutamate (green) in mCherry-labeled neurons (red) in chemogenetic virus-injected mice. Scale bars: 100 μm (left), 2 μm, (right). ***C***, The colocalization rate of mCherry and glutamate (*n* = 15 slices/group; *N* = 3 mice/group). The data are presented as the means ± SEMs. ***D***, Representative immunofluorescence image showing the localization of vglut2 (green) in mCherry-labeled neurons (red). Scale bar, 20 μm. ***E***, The colocalization rate of mCherry and vglut2 (*n* = 15 slices/group; *N* = 3 mice/group). The data are presented as the means ± SEMs. ***F***, Schematic diagram of chemogenetic virus injection and whole-cell recording in C57BL/6 mice. ***G***, Representative traces of evoked responses to 100 and 200 pA current injections in the sham (blue) group and SNI (red) group. ***H***, Average number of induced spikes in vlPAG pyramidal neurons in response to step current injections in the sham (blue) group and SNI (red) group. *n* = 10 neurons/group; *N* = 3–4 mice/group. ***p < 0.001 versus the sham group. ***I***, Rheobase values for the sham (blue) and SNI (red) model mice. *n* = 10 neurons/group; *N* = 3–4 mice/group. *p < 0.05. ***J***, Representative immunofluorescence image of glutamate and c-Fos colocalization in the vlPAG. Scale bar, 200 μm. ***K***, Colocalization rate of c-Fos and glutamate. *n* = 15 slices/group; *N* = 3 mice/group. The mean difference between the sham and SNI groups is shown in the above Cumming estimation plots. The raw data are plotted on the left axis; the mean difference is plotted on the floating axis on the right as a bootstrap sampling distribution. The mean differences are shown as dots; the 95% confidence intervals are indicated by the vertical error bars.

SNI model mice exhibit allodynia accompanied by anxiety-like behaviors, and the vlPAG is related to pain and negative emotions. However, the specific neural cells involved in the regulation of pain remain unknown. In the following experiments, we used a chemogenetic virus labeled with mCherry to specifically label vlPAG neurons (Fig. 2*A*). Approximately 80% of vlPAG neurons labeled with mCherry were immunoreactive for glutamate, and 83% were immunoreactive for vesicular glutamate transporter 2 (vGLUT2; Rossi et al., 2021), indicating that these neurons were CaMKIIα^+^ neurons (Fig. 2*B–E*). Next, we evaluated the activity of vlPAG^CaMKIIα+^ neurons in each group of mice. The virus (AAV2/9-CaMKIIα-mCherry) was injected into the right vlPAG 14 d before modeling. Whole-cell recording showed that the number of action potentials produced by vlPAG^CaMKIIα+^ neurons was significantly reduced in SNI model mice compared with the sham group (two-way RM ANOVA: *F*_(10,209) _= 15.64; *p *< 0.001; Tukey's post hoc test: *p *< 0.001; Fig. 2*F–H*). The rheobase was greater in the SNI group than that in the sham group (*t* = 3.502; mean difference = 29.0 [95.0% CI 12.0, 43.0]; *p *< 0.01; Fig. 2*I*). In addition, we used immunofluorescence to assess the activation of pyramidal neurons. The immediate early gene c-Fos is rapidly expressed in neurons after exogenous stimulation and serves as a marker of neuronal activation (Joo et al., 2016). To evaluate the activation of pyramidal neurons, we measured the colocalization rate between glutamate and c-Fos. The colocalization rate in SNI model mice was significantly lower than that in sham group mice (*t* = 8.891; mean difference = −22.6 [95.0% CI −27.5, −17.9]; *p *< 0.001; Fig. 2*J*,*K*). These results suggest that the excitability of vlPAG^CaMKIIα+^ neurons was decreased in SNI model mice.

### Activation of vlPAG^CaMKIIα+^ neurons alleviated allodynia and anxiety-like behaviors in SNI model mice

**Figure 3.**
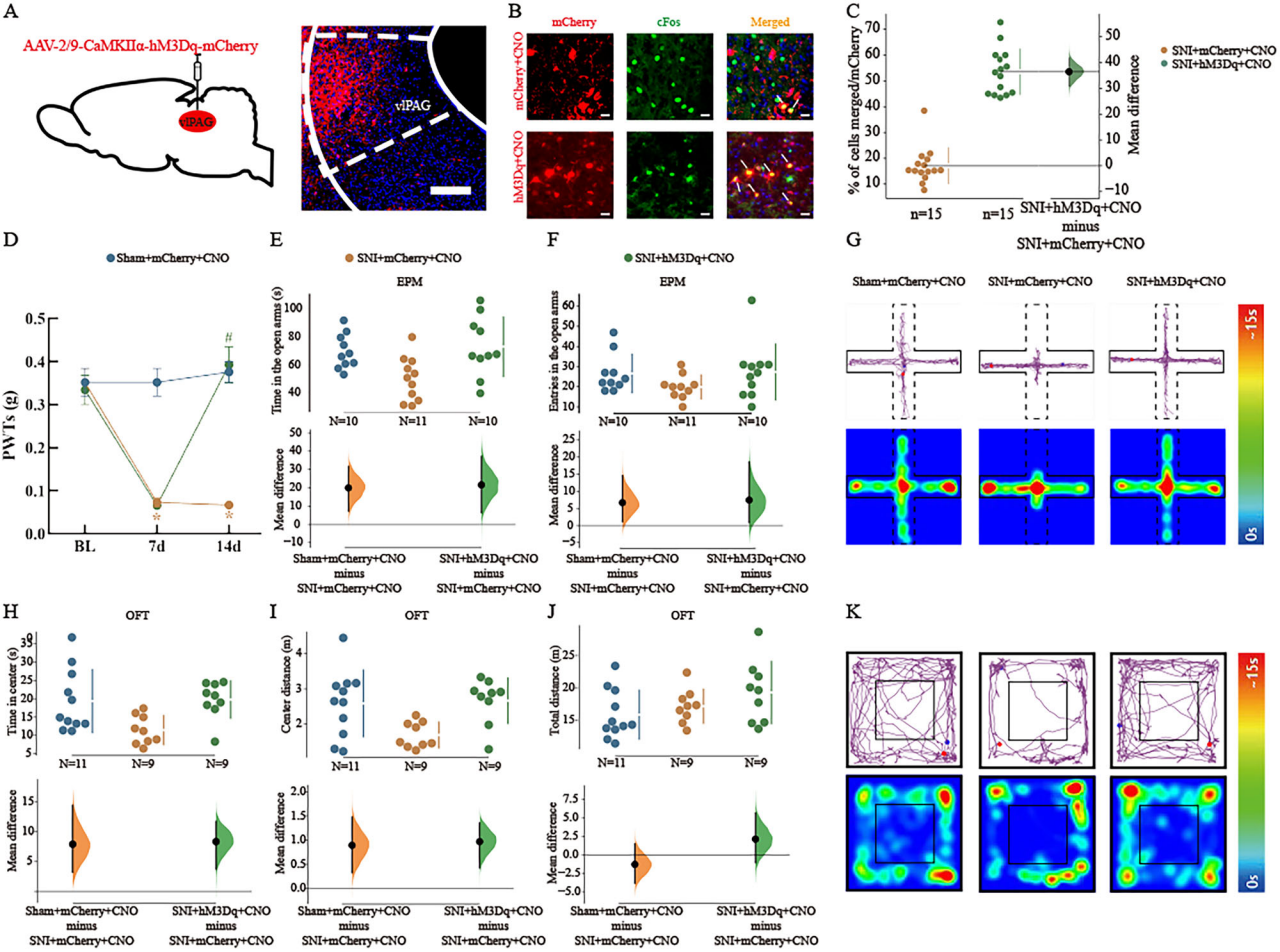
Activation of vlPAG^CaMKIIα+^ neurons alleviated SNI-induced allodynia and anxiety-like behaviors. ***A***, Schematic diagram of chemogenetic virus injection into C57BL/6 mice and representative coronal sections showing virus infection in the vlPAG. Scale bar, 100 μm. ***B***, Representative immunofluorescence images showing the localization of c-Fos (green) in mCherry-labeled neurons (red) in the vlPAG. Scale bar, 20 μm. ***C***, Colocalization rate of mCherry and c-Fos. *n* = 15 slices/group; *N* = 3 mice/group. The raw data are plotted on the left axis; the mean difference is plotted on the floating axis on the right as a bootstrap sampling distribution. The mean differences are shown as dots; the 95% confidence intervals are indicated by the vertical error bars. ***D***, Mechanical PWTs after chemogenetic regulation of vlPAG^CaMKIIα+^ neurons following CNO administration. *N* = 10–11 per group. The data are presented as the means ± SEMs. *p < 0.05 versus the sham + mCherry + CNO group; ^#^*p *< 0.05 versus the SNI + mCherry + CNO group. ***E***, ***F***, Quantitative analysis of the time spent in the open arms and the number of entries into the open arms in the EPM after activation of vlPAG^CaMKIIα+^ neurons. *N* = 10–11 per group. ***G***, Representative diagrams showing the movement trajectories and activity heatmaps of the three groups in the EPM. ***H–J***, Quantitative analysis of time spent in the center, distance traveled in the center, and total distance traveled in the OFT after activation of vlPAG^CaMKIIα+^ neurons. *N* = 9–11 per group. ***K***, Representative diagrams showing the movement trajectories and activity heatmaps of the three groups in the OFT. The mean differences for the three groups are shown in the above Cumming estimation plot. The raw data are plotted on the top axes. On the bottom axis, the mean differences are plotted as bootstrap sampling distributions. The mean differences are shown as dots. The 95% confidence intervals are indicated by the vertical error bars.

We found that the activity of vlPAG^CaMKIIα+^ neurons was decreased in SNI model mice. To investigate whether vlPAG^CaMKIIα+^ neurons regulate chronic neuropathic-induced anxiety, we injected a Cre-dependent activation virus (AAV2/9-CamKⅡα-hM3Dq-mCherry) into the right vlPAG of SNI model mice to specifically activate glutamatergic neurons and evaluate the effects of these neurons on the mechanical pain threshold and anxiety-like behaviors. A schematic of this experiment is shown in Figure 3*A*. Three weeks after virus injection, we observed CaMKIIα^+^ neurons in the vlPAG (Fig. 3*B*). We also examined the activity of vlPAG^CaMKIIα+^ neurons. The colocalization of CaMKIIα^+^ with c-Fos (*t* = 12.53; mean difference = 36.4 [95.0% CI 30.8, 41.8]; *p *< 0.01; Fig. 3*C*) confirmed that the hM3Dq virus was able to activate CaMKIIα^+^ neurons. At 7 and 14 d after SNI, the PWT was significantly lower in the SNI + mCherry + CNO group than that in the sham + mCherry + CNO group (two-way RM ANOVA: *F*_(2,84) _= 33.52; *p *< 0.001; Tukey's post hoc test: *p *< 0.001; Fig. 3*D*). After injection of CNO, the PWT was significantly greater in the SNI + hM3Dq + CNO group than that in the SNI + mCherry + CNO group at 14 d (followed by Tukey's post hoc test: *p *< 0.001; Fig. 3*D*). We found that the SNI + mCherry + CNO group spent less time in the open arms of the EPM than the sham + mCherry + CNO group did (one-way ANOVA: *F*_(2,28) _= 5.041; *p *< 0.05; Tukey's post hoc test: mean difference = 20.0 [95.0% CI 7.27, 31.7]; *p *< 0.05; Fig. 3*E*). However, the SNI + hM3Dq + CNO group spent more time in the open arms of the EPM (Tukey's post hoc test: mean difference = 21.6 [95.0% CI 6.36, 37.2]; *p *< 0.05; Fig. 3*E*). There was no significant difference in the number of entries into the open arms of the EPM among the three groups (one-way ANOVA: *F*_(2,28) _= 1.574; *p *> 0.05; Fig. 3*F*). The SNI + mCherry + CNO group spent less time traveling a shorter distance in the center of the open field than the sham + mCherry + CNO group. The SNI + hM3Dq + CNO group spent more time and traveled farther into the center of the open field than did the SNI + mCherry + CNO group (one-way ANOVA: *F*_(2,26) _= 4.983; *p *< 0.05; Tukey's post hoc test: mean difference = 7.92 [95.0% CI 3.22, 14.4]; *p *< 0.05; mean difference = 8.36 [95.0% CI 3.74, 11.7]; *p *< 0.05; Fig. 3*H*; one-way ANOVA: *F*_(2,26) _= 5.290, *p *< 0.05; Tukey's post hoc test: mean difference = 0.894 [95.0% CI 0.326, 1.48]; *p *< 0.05; mean difference = 0.971 [95.0% CI 0.427, 1.36]; *p *< 0.05; Fig. 3*I*). The total distance traveled in the OFT was not significantly different among the three groups (Fig. 3*J*; one-way ANOVA: *F*_(2,26) _= 1.987; *p *> 0.05). Representative diagrams showing the movement trajectories and activity heatmaps of the three groups in the EPM and OFT are shown in Figure 3*G*,*K*, respectively. These results suggest that activation of vlPAG^CaMKIIα+^ neurons could alleviate allodynia and anxiety-like behaviors induced by SNI.

### EA effectively alleviated allodynia and anxiety-like behaviors induced by SNI and activated vlPAG^CaMKIIα+^ neurons

**Figure 4.**
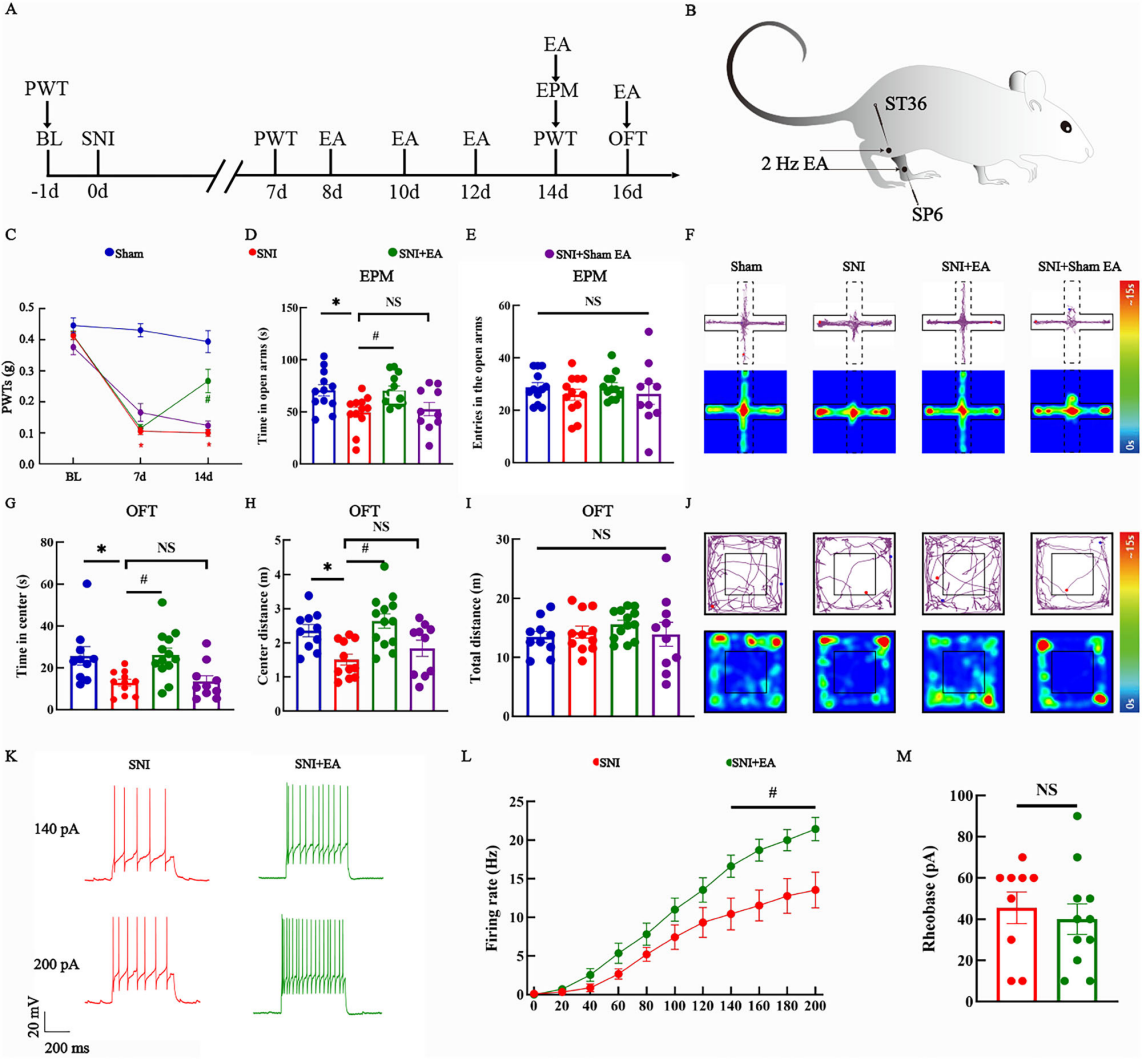
EA alleviated SNI-induced allodynia and anxiety-like behaviors. ***A***, Schematic diagram of the experimental process. ***B***, Schematic of the EA treatment protocol. ***C***, Mechanical PWTs after SNI and EA treatment. The data are presented as the means ± SEMs. *N* = 13–15 per group. *p < 0.05; ^#^*p *< 0.05. ***D***, ***E***, Quantitative analysis of the time spent in the open arms and number of entries into the open arms by the four groups of mice in the EPM test. *N* = 10–12 per group. *p < 0.05; ^#^*p *< 0.05; NS, not significant. ***F***, Representative diagrams showing the movement trajectories and activity heatmaps of the four groups in the EPM. ***G–I***, Quantitative analysis of time spent in the center, distance traveled in the center, and total distance traveled during the OFT. *N* = 10–13 per group. *p < 0.05; ^#^*p *< 0.05; NS, not significant. ***J***, Representative diagrams showing the movement trajectories and activity heatmaps of the four groups in the OFT. ***K***, Representative traces of evoked responses to 140 and 200 pA current injections in the SNI and SNI + EA groups. ***L***, Average number of induced spikes in vlPAG^CaMKIIα+^ neurons in response to step current injections in the SNI and SNI + EA groups. *n* = 9–11 neurons/group; *N* = 3–4 mice/group. ^#^*p *< 0.05. ***M***, Rheobase values recorded for vlPAG^CaMKIIα+^ neurons in SNI and SNI + EA mice. *n* = 9–11 neurons/group; *N* = 3–4 mice/group. NS, not significant. The data are presented as the means ± SEMs. *p < 0.05 versus the sham group; ^#^*p *< 0.05 versus the SNI group.

In the previous part of the experiment, we constructed a model of SNI-induced chronic neuropathic pain. In this part, we investigated the effect of EA treatment on SNI model mice. The experimental process is shown in Figure 4*A*. Zusanli (ST36) and Sanyinjiao (SP6) were selected as the acupoints for EA treatment (Fig. 4*B*). Starting 8 d after SNI, the mice in the SNI + EA group received EA treatment (2 Hz, 0.1 mA, 30 min) every 2 d. In the SNI + sham EA group, needles were inserted into the SP6 and ST36 acupoints, but no current was delivered. The PWT of the left hindpaw before SNI was not significantly different among the four groups. At 7 d after SNI, the PWTs of the SNI group, SNI + EA group, and SNI + sham EA group were significantly lower than those of the sham group (two-way ANOVA: *F*_(2,144) _= 106.5; *p *< 0.05; Tukey's post hoc test: *p *< 0.05; Fig. 4*C*). On the 14th day after SNI, compared with those of mice in the sham group, the PWTs of mice in the SNI group were significantly lower (Tukey's post hoc test: *p *< 0.05; Fig. 4*C*). Compared with those of mice in the SNI group and SNI + sham EA group, the PWTs of mice in the SNI + EA group were significantly greater (Tukey's post hoc test: *p *< 0.05; Fig. 4*C*). EA treatment significantly increased the PWTs of SNI model mice from Days 8 to 14. However, SNI model mice in the sham EA group did not exhibit increased PWTs (Fig. 4*C*). We found that the SNI group spent less time in the open arms of the EPM than the sham group did. The time spent in the open arms of the EPM was increased in the SNI + EA group but not in the SNI + sham EA group (one-way ANOVA: *F*_(3,42) _= 4.750; *p *< 0.05; Tukey's post hoc test: *p *< 0.05; Fig. 4*D*). There was no significant difference in the number of entries into the open arms of the EPM among the four groups (one-way ANOVA: *F*_(3,42) _= 0.507; *p *> 0.05; Fig. 4*E*). The SNI group spent less time and traveled a shorter distance in the center of the open field than the sham group. The SNI + EA group spent more time and traveled a farther distance in the center of the open field than the SNI group, but the SNI + sham + EA group did not (one-way ANOVA: *F*_(3,40) _= 5.735; *p *< 0.05; Tukey's post hoc test: *p *< 0.05; Fig.4*G*; one-way ANOVA: *F*_(3,42) _= 6.713; *p *< 0.05; Tukey's post hoc test: *p *< 0.05; Fig. 4*H*). The total distance traveled in the open field was not significantly different among the four groups (one-way ANOVA: *F*_(3,42) _= 0.610; *p *> 0.05; Fig. 4*I*). Representative diagrams showing the movement trajectories and activity heatmaps of the four groups in the EPM and OFT are shown in Figure 4*F*,*J*, respectively. Then, we investigated the excitability of vlPAG^CaMKIIα+^ neurons by whole-cell recording and found that the number of action potentials was significantly greater in the SNI + EA group than that in the SNI group (two-way ANOVA: *F*_(10,198) _= 43.19; *p *< 0.05; Tukey’s post hoc test's: *p *< 0.05; Fig. 4*K*,*L*). The rheobase was not greater in the SNI + EA group than that in the SNI group (*t* = 0.501; *p *> 0.05; Fig. 4*M*), indicating that the effect of EA on vlPAG^CaMKIIα+^ neurons was presynaptic. These results indicate that EA, but not sham EA, significantly alleviated SNI-induced allodynia and anxiety-like behaviors. In addition, EA activated vlPAG^CaMKIIα+^ neurons.

### Inhibition of vlPAG^CaMKIIα+^ neurons induced allodynia and anxiety-like behaviors in naive mice and abolished the effects of EA intervention

**Figure 5.**
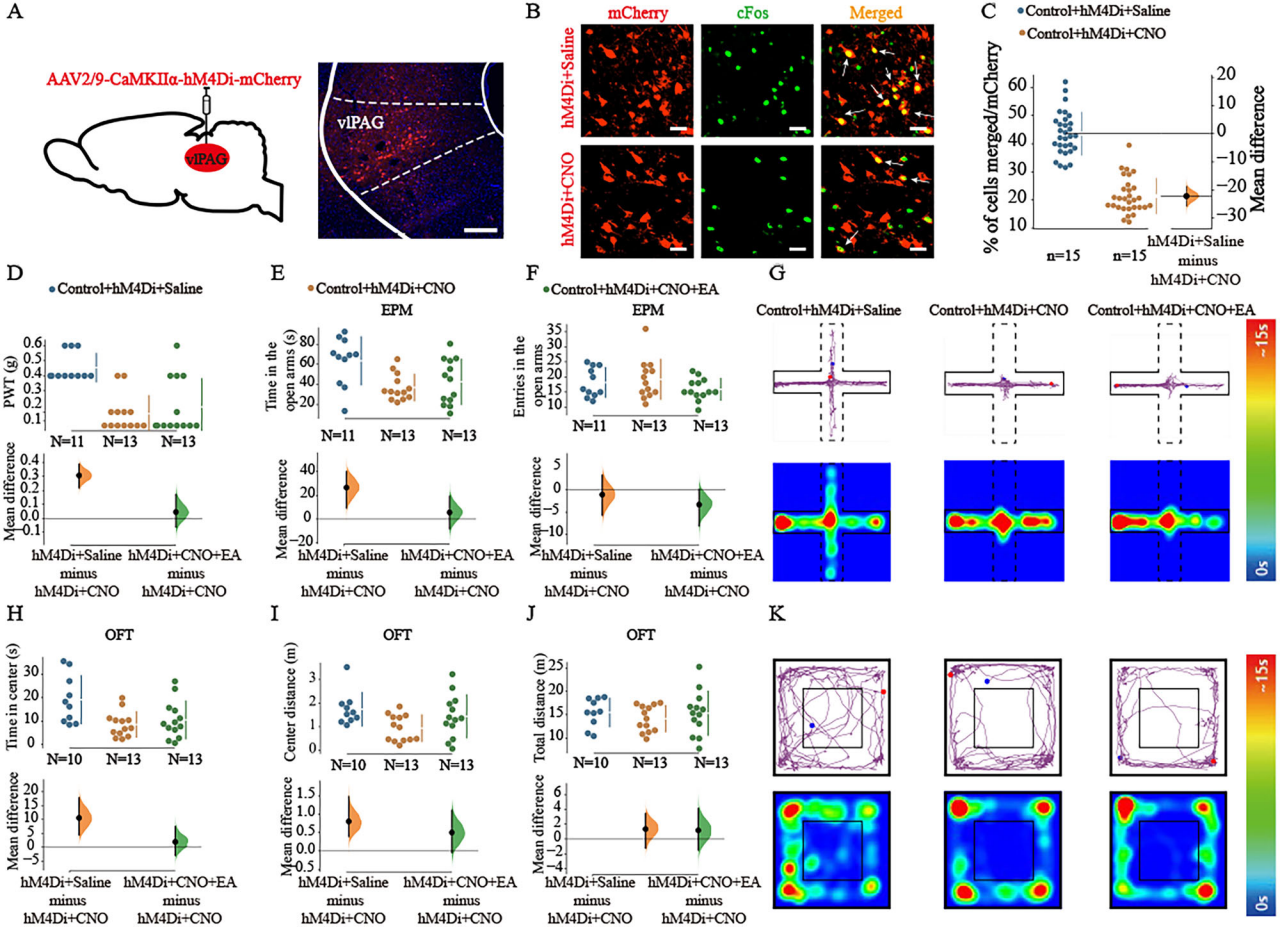
Inhibition of vlPAG^CaMKIIα+^ neurons led to allodynia and anxiety-like behaviors in naive mice and abolished the effects of EA intervention. ***A***, Schematic representation of vlPAG injection of the hM4Di virus into C57BL/6 mice (left) and representative coronal sections showing virus infection in the vlPAG (right). Scale bar, 100 μm. ***B***, Representative immunofluorescence images showing the localization of c-Fos in mCherry-labeled neurons. Scale bar, 20 μm. ***C***, Colocalization rate of mCherry and c-Fos. *n* = 15 slices/group; *N* = 3–4 mice/group. The raw data are plotted on the left axis; the mean difference is plotted on the floating axis on the right as a bootstrap sampling distribution. The mean differences are shown as dots; the 95% confidence intervals are indicated by the vertical error bars. ***D***, The mechanical PWT of each group after chemogenetic inhibition of vlPAG^CaMKIIα+^ neurons following CNO administration. *N* = 11–13 per group. ***E***, ***F***, Quantitative analysis of the time spent in the open arms and the number of entries into the open arms in the EPM after the inhibition of vlPAG^CaMKIIα+^ neurons. *N* = 11–13 per group. ***G***, Representative diagrams showing the movement trajectories and activity heatmaps of the three groups in the EPM. ***H–J***, Quantitative results of the time spent in the center, distance traveled in the center ,and total distance traveled in the OFT after the inhibition of vlPAG^CaMKIIα+^ neurons. *N* = 10–13 per group. ***K***, Representative diagrams showing the movement trajectories and activity heatmaps of the three groups in the OFT. The mean differences for the three groups are shown in the above Cumming estimation plot. The raw data are plotted on the top axes. On the bottom axis, the mean differences are plotted as bootstrap sampling distributions. The mean differences are shown as dots. The 95% confidence intervals are indicated by the vertical error bars.

For the subsequent experiment, we injected a Cre-dependent activation virus (AAV2/9-CamKⅡα-hM4Di-mCherry) into the bilateral vlPAG of naive mice to investigate whether the inhibition of vlPAG^CaMKIIα+^ neurons regulated anxiety-like behaviors as well as pain and the effect of EA. Three weeks after virus injection, we found CaMKIIα^+^ neurons in the vlPAG (Fig. 5*A*). We also examined whether the virus inhibited CaMKIIα^+^ neuron activity. The reduction in the colocalization of CaMKIIα^+^ with c-Fos (Fig. 5*B*) confirmed that the hM4Di virus was able to inhibit CaMKIIα^+^ neuron activity (*t* = 12.25; mean difference = −22.2 [95.0% CI −25.8, −18.8]; *p *< 0.05; Fig. 5*C*). The PWT was significantly lower in the control + hM4Di + CNO group than that in the control + hM4Di + saline group. However, there was no significant difference in PWT between the control + hM4Di + CNO group and the control + hM4Di + CNO + EA group (one-way ANOVA: *F*_(2,34) _= 16.00; *p *< 0.05; Tukey's post hoc test: mean difference = 0.306 [95.0% CI 0.219, 0.386]; *p *< 0.05; mean difference = 0.0454 [95.0% CI −0.0623, 0.171]; *p *> 0.05; Fig. 5*D*). We found that the control + hM4Di + CNO group spent less time in the open arms of the EPM than the control + hM4Di + saline group did, but there was no significant difference between the control + hM4Di + CNO group and the control + hM4Di + CNO + EA group (one-way ANOVA: *F*_(2,34) _= 5.590; *p *< 0.001; Tukey's post hoc test: mean difference = 26.6 [95.0% CI 9.37, 39.8]; *p *< 0.05; mean difference = 5.68 [95.0% CI −8.18, 19.3]; *p *> 0.05; Fig. 5*E*). Moreover, there was no significant difference in the number of entries into the open arms of the EPM among the three groups (one-way ANOVA: *F*_(2,34) _= 1.368; *p *> 0.05; mean difference = −1.05 [95.0% CI −5.73, 3.27]; mean difference = −3.31 [95.0% CI −8.15, 0.154]; Fig. 5*F*). The control + hM4Di + CNO group spent less time and traveled a shorter distance in the center of the open field than the control + hM4Di + saline group did, but there was no significant difference between the control + hM4Di + CNO group and the control + hM4Di + CNO + EA group (one-way ANOVA: *F*_(2,33)_ = 5.516; *p *< 0.05; Tukey's post hoc test; mean difference = 10.6 [95.0% CI 4.44, 17.8]; *p *< 0.05; mean difference = 1.95 [95.0% CI −2.96, 7.42]; *p *> 0.05; Fig. 5*H*; one-way ANOVA: *F*_(2,33)_ = 3.489; *p *< 0.05; Tukey's post hoc test; mean difference = 0.803 [95.0% CI 0.394, 1.47]; *p *< 0.05; mean difference = 0.502 [95.0% CI −0.0438, 1.1], *p *> 0.05; Fig. 5*I*). The total distance traveled in the OFT was not significantly different among the three groups (one-way ANOVA: *F*_(2,33) _= 0.484; *p *> 0.05; Fig. 5*J*). Representative diagrams showing the movement trajectories and activity heatmaps of the three groups in the EPM and OFT are shown in Figure 5*G*,*K*, respectively. Our work suggests that the inhibition of vlPAG^CaMKIIα+^ neurons led to allodynia and anxiety-like behaviors similar to those induced by SNI and abolished the analgesic and anxiolytic effects of EA treatment.

### EA treatment combined with chemogenetic activation of vlPAG^CaMKIIα+^ neurons failed to exhibit enhanced analgesic and antianxiety-like effects

**Figure 6.**
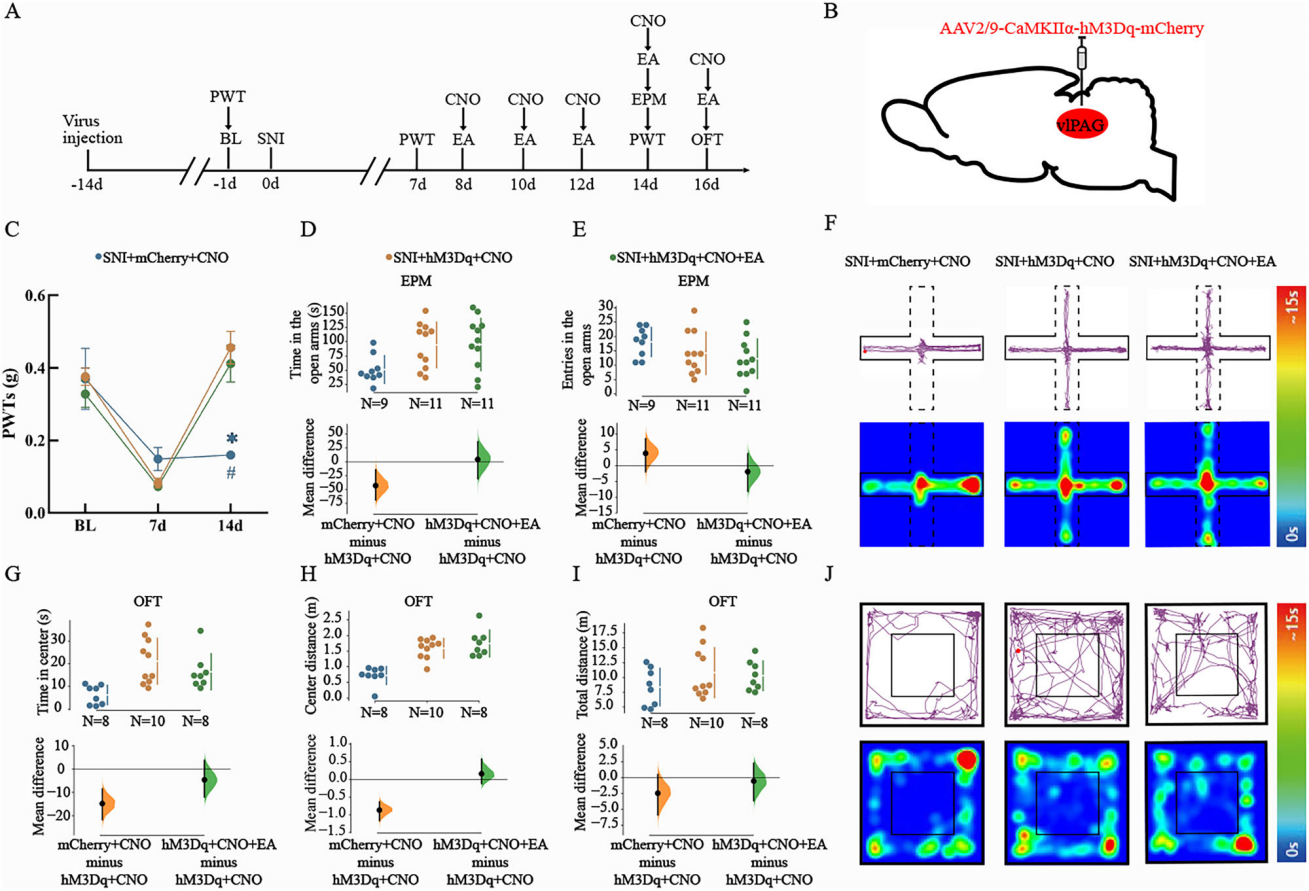
Activation of vlPAG^CaMKIIα+^ neurons underlies the EA-mediated alleviation of allodynia and anxiety-like behaviors. ***A***, Schematic diagram of the experimental process. ***B***, Schematic diagram of the injection of the hM3Dq virus into C57BL/6 mice. ***C***, Mechanical PWTs after chemogenetic activation of vlPAG^CaMKIIα+^ neurons following CNO administration. *N* = 9–11 per group. The data are presented as the means ± SEMs. *p < 0.05 versus the SNI + hM3Dq + CNO group; ^#^*p *< 0.05 versus the SNI + hM3Dq + CNO + EA group. ***D***, ***E***, Quantitative results of the time spent in the open arms (***D***) and number of entries into the open arms (***E***) in the EPM after activation of vlPAG^CaMKIIα+^ neurons. *N* = 9–11 per group. ***F***, Representative diagrams showing the movement trajectories and activity heatmaps of the three groups in the EPM. ***G–I***, Quantitative analysis of the time spent in the center (***G***), distance traveled in the center (***H***), and total distance traveled (***I***) in the OFT after activation of vlPAG^CaMKIIα+^ neurons. *N* = 8–10 per group. ***J***, Representative diagrams showing the movement trajectories and activity heatmaps of the three groups in the OFT. The mean differences for the three groups are shown in the above Cumming estimation plot. The raw data are plotted on the top axes. On the bottom axis, the mean differences are plotted as bootstrap sampling distributions. The mean differences are shown as dots. The 95% confidence intervals are indicated by the vertical error bars.

The above experimental results indicated that the activation of vlPAG^CaMKIIα+^ neurons could alleviate allodynia and anxiety-like behaviors in SNI model mice and that EA had a similar effect. Therefore, under pathological conditions, we specifically activated vlPAG^CaMKIIα+^ neurons and administered EA at the same time to assess whether EA regulates pain through vlPAG^CaMKIIα+^ neurons. The procedure used in this part of the experiment is shown in Figure 6*A*. We injected a virus (AAV2/9-CamKⅡα-hM3Dq-mCherry) into the right vlPAG of SNI model mice (Fig. 6*B*). We found that at 7 d, the PWT did not significantly differ between the groups. Fourteen days after CNO injection, the PWT was significantly greater in the SNI + hM3Dq + CNO group and SNI + hM3Dq + CNO + EA group than that in the SNI + mCherry + CNO group (two-way ANOVA: *F*_(2,75) _= 63.66; *p *< 0.05; Tukey’ post hoc test: *p *< 0.05; *p *< 0.05; Fig. 6*C*). We found that the SNI + hM3Dq + CNO group and SNI + hM3Dq + CNO + EA group spent more time in the open arms of the EPM than did the SNI + mCherry + CNO group (one-way ANOVA: *F*_(2,28) _= 4.392; *p *< 0.05; Tukey's post hoc test: mean difference = −42.95 [95.0% CI −68.56, −14.59]; *p *< 0.05; mean difference = 3.95 [95.0% CI −30.67, 35.6]; *p *< 0.05; Fig. 6*D*). There was no significant difference in the number of entries into the open arms of the EPM among the three groups (one-way ANOVA: *F*_(2,28) _= 1.974; *p *> 0.05; mean difference = 3.93 [95.0% CI −1.91, 8.55]; *p *> 0.05; mean difference = −1.91 [95.0% CI −8.09, 3.82]; Fig. 6*E*). The SNI + hM3Dq + CNO group and SNI + hM3Dq + CNO + EA group spent more time and traveled a shorter distance in the center of the open field than did the SNI + mCherry + CNO group (one-way ANOVA: *F*_(2,23) _= 7.752; *p *< 0.05; Tukey's post hoc test: mean difference = −14.9 [95.0% CI −21.8, −8.54]; *p *< 0.05; mean difference = −4.67 [95.0% CI −12.0, 3.65]; *p *< 0.05; Fig. 6*G*; one-way ANOVA: *F*_(2,28) _= 20.74; *p *< 0.05; Tukey's post hoc test: mean difference = −0.871 [95.0% CI −1.17, −0.636]; *p *< 0.05; mean difference = 0.157 [95.0% CI −0.119, 0.572]; *p *< 0.05; Fig. 6*H*). The total distance traveled in the OFT was not significantly different among the three groups (one-way ANOVA: *F*_(2,23) _= 1.177; *p *< 0.05; mean difference = −2.43 [95.0% CI −5.86, 0.529]; mean difference = −0.518 [95.0% CI −3.61, 2.3]; Fig. 6*I*). Representative diagrams showing the movement trajectories and activity heatmaps of the three groups in the EPM and OFT are shown in Figure 6*F*,*J*, respectively. Interestingly, there was no significant difference in PWT or anxiety-like behaviors between the SNI + hM3Dq + CNO group and the SNI + hM3Dq + CNO + EA group. These results indicate that EA treatment combined with chemogenetic activation of vlPAG^CaMKIIα+^ neurons failed to further alleviate the reduction in the mechanical pain threshold and anxiety-like behaviors in SNI model mice. We speculate that EA exerts analgesic and anxiolytic effects by activating vlPAG^CaMKIIα+^ neurons.

## Discussion

The periaqueductal gray is thought to play an essential role in the descending pain modulatory system, but the neuronal type that is related to chronic neuropathic pain-induced anxiety has not yet been revealed. Here, in an animal model of SNI, we obtained evidence via immunofluorescence and whole-cell recordings that neuropathic pain deactivated vlPAG^CaMKIIα+^ neurons. Increasing the excitability of CaMKIIα^+^ neurons could alleviate SNI-induced allodynia and anxiety-like behaviors, while inhibiting these neurons may exacerbate these symptoms. EA treatment could relieve SNI-induced algesia and anxiety-like behaviors, directly increasing vlPAG^CaMKIIα+^ neuron excitability in SNI model mice. Our data suggest that the firing rate of vlPAG^CaMKIIα+^ neurons was increased in the SNI + EA group. Artificial inhibition of vlPAG^CaMKIIα+^ neurons abolished the anxiolytic and analgesic effects of EA, and artificial activation of vlPAG^CaMKIIα+^ neurons combined with EA treatment did not exert synergistic effects. Hence, we hypothesized that EA may exert effects against pain and negative emotions through the activation of vlPAG^CaMKIIα+^ neurons. These results elucidate the importance of precise interventions for comorbid pain and anxiety.

As we previously mentioned, the vlPAG contains various neurotransmitters, including glutamate, gamma-aminobutyric acid, and serotonin, which are closely associated with pain regulation (Descalzi et al., 2017). Recent studies have also shown that the expression level of glutamate in the vlPAG is decreased in pain model mice (Tůma et al., 2013), and other researchers have noted that selective optogenetic activation of vlPAG glutamatergic neurons increases the mechanical pain threshold in normal mice (Tovote et al., 2016). In addition, the activation of astrocytes in the vlPAG has a regulatory effect on the pain response (Liu et al., 2022). Specific activation of cannabinoid receptors on vlPAG glutamatergic neurons can produce analgesic effects (H. Zhu et al., 2019). Remarkably, when vlPAG glutamatergic neurons are selectively activated, the degree of analgesia increases significantly (Liu et al., 2022). Our results also revealed that increased activation of vlPAG^CaMKIIα+^ neurons could exert analgesic effects. These findings suggest that vlPAG^CaMKIIα+^ neurons are vital for analgesic effects.

Although vlPAG glutamatergic neurons were previously found to be key for pain regulation (Bagley and Ingram, 2020), the vlPAG has also been reported to be associated with anxiety (Vázquez-León et al., 2022). The T1-weighted manganese-enhanced magnetic resonance imaging signal intensity was found to be increased in supraspinal regions of the aversion and anxiety circuitry, including the anterior cingulate gyrus and vlPAG (McIlwrath et al., 2020). Astrocytes in the vlPAG regulate diabetes-associated neuropathic pain and concomitant anxiety-like behavior, as reported by L. Yang et al. (2022). Psychological stress-elicited mental disorders are related to a marked decrease in glutamatergic transmission in the vlPAG (Peng et al., 2022). Many genes have been implicated in anxiety, depression, and chronic pain in patients. Giannina Descalzi reported on the changes in gene expression induced by neuropathic pain in three distinct brain regions (the nucleus accumbens, medial prefrontal cortex, and ventral lateral periaqueductal gray) and revealed molecular connections between pain and chronic stress (Descalzi et al., 2017). Vázquez-León et al. (2021) suggested that the vlPAG contributes to the modulation of anxiety, fear, and nociception linked with chronic exposure to drugs of abuse. A reduction in glutamate receptor 1-associated signaling on the cell surface and in the cytosol in the vlPAG contributes to chronic stress-induced neuroplastic changes and may play a critical role in the pathogenesis of stress-associated neuropsychiatric disorders (Y. C. Ho et al., 2018). Although the vlPAG was reported to be independently associated with pain and anxiety, its role in chronic neuropathic pain-induced anxiety has not been elucidated. Unlike the findings of other studies, our study showed that selectively manipulating vlPAG^CaMKIIα+^ neurons affects not only allodynia but also anxiety-like behaviors.

In this investigation, we found that 2 Hz EA produced robust analgesic and anxiolytic effects, as previously reported (M. Wu et al., 2022). EA decreases the levels of inflammatory mediators in the prefrontal cortex, hypothalamus, vlPAG, and other brain regions to relieve chronic pain and accompanying negative emotions (Liao and Lin, 2021). EA was found to regulate anxiety-like behaviors in CFA-treated rats through the rACC→thalamus circuit (Shen et al., 2020). The analgesic effect of EA on neuropathic pain may be related to the suppression of glucose metabolism and glucose transporter 3 expression (Jiang et al., 2021). In this study, we proved that EA may exert analgesic and anxiolytic effects by enhancing vlPAG^CaMKIIα+^ neuron activity. In the present work, enhancing vlPAG^CaMKIIα+^ neuron activity relieved pain and anxiety-like behaviors. However, our previous studies showed that activation of the rACC^Glu^→vlPAG circuit could induce algesia and anxiety-like behaviors in mice, and inhibition of the rACC^Glu^→vlPAG circuit could alleviate algesia and anxiety-like behaviors in SNI model mice (X. Zhu et al., 2021). The most likely explanation is that glutamatergic projections from the rACC directly control GABAergic neurons in the vlPAG, and existing monosynaptic connections between the rACC^Glu^→vlPAG^GABA^ circuit and GABAergic neurons can suppress glutamatergic neuron activity locally. It is reasonable to assume that there is a rACC^Glu^→vlPAG^GABA^→vlPAG^Glu^ microcircuit involving feedforward inhibition. This complex mechanism needs to be further investigated.

On the one hand, our previous laboratory results showed that activation of the rACC^Glu^→vlPAG circuit could antagonize the analgesic effect but not the anxiolytic effect of EA. On the other hand, in the present study, EA may regulate both pain and chronic neuropathic pain-induced anxiety-like behaviors via vlPAG^CaMKIIα+^ neurons. This difference may be related to the simultaneous regulation of vlPAG activity by different upstream brain regions (Huang et al., 2019; Takeoka and Arber, 2019). According to Hu et al. (2022), bright light treatment suppresses mouse nociceptive behaviors through a visual circuit related to the lateral periaqueductal gray and vlPAG. Other researchers have reported that the vlPAG receives projections from the lateral hypothalamus. In two models of persistent pain, optogenetic activation of lateral hypothalamic parvalbumin neurons or their vlPAG axonal projections was found to attenuate nociception, and neuroanatomical tracing revealed that lateral hypothalamic parvalbumin neurons preferentially target glutamatergic neurons over GABAergic neurons in the vlPAG (Siemian et al., 2021). Furthermore, several researchers have suggested that the thalamic paraventricular nucleus→central amygdala→vlPAG circuit mediates central mechanisms of descending pain facilitation underlying persistent pain conditions (Liang et al., 2020). As a key brain region in the descending pain modulatory system, the vlPAG may be controlled by multiple brain regions simultaneously, thus exerting analgesic and antianxiety effects. We speculate that EA may also contribute to alleviating neuropathic pain-induced anxiety by simultaneously regulating vlPAG activity through multiple circuits. Moreover, our whole-cell recording results suggested that EA may alter the excitability of vlPAG^CaMKIIα+^ neurons through presynaptic effects, providing evidence for the regulatory role of EA in multiple circuits.

Overall, we demonstrated that increasing the excitability of glutamatergic neurons could relieve SNI-induced allodynia and anxiety-like behaviors, while inhibiting these neurons induced these symptoms. In particular, EA may exert anxiolytic and analgesic effects by increasing the excitability of vlPAG^CaMKIIα+^ neurons. This study focused on the effect of vlPAG^CaMKIIα+^ neurons on pain and emotion. These results provide additional evidence for the further study of microcircuits, which may be important for understanding the neural mechanism of neuropathic pain and anxiety, the management of which remains a major medical challenge (Hooten, 2016; Kühlmann et al., 2018).

## Data Availability

Data are available from the corresponding author without undue reservation.

## Synthesis

Reviewing Editor: Christophe Bernard, INSERM &Institut de Neurosciences des Systèmes Decisions are customarily a result of the Reviewing Editor and the peer reviewers coming together and discussing their recommendations until a consensus is reached. When revisions are invited, a fact-based synthesis statement explaining their decision and outlining what is needed to prepare a revision will be listed below. The following reviewer(s) agreed to reveal their identity: Thomas Kash.

After consultation between your reviewers and reviewing editors, please find the synthesis of our comments:

1) You are using a camk2 driven aav to transduce neurons in the vPAG and examine impact on SNI and EA related behaviors. You evaluate how effective the virus is by costaining with glutamate. This is not an appropriate validation, as glutamate can be present in different neurons. For your interpretation to hold water, you need to use a more detailed molecular approach to determine what cell types this virus expresses in, such as in situ. This is critical, as recent studies have found camk2-driven viruses can infect interneurons, and camk2 is highly expressed in gaba projection neurons, as well as medium spiny neurons.

2) How can you explain your findings in light with the previous contradicting findings (in Zhu. Et al 2021) that the activation of glutamatergic projections from the rostral anterior cingulate cortex (rACCGlu) to the ventrolateral periaqueductal gray (vlPAG) have shown to induce both hyperalgesia and anxiety-like behaviors. Also, EA effectively alleviated these symptoms via suppression of the neuronal activation. Although cited in the discussion, these contradicting findings are not fully justified. Perhaps more experiments (other than that used in Zhu et al 2021) are needed to validate neuronal activation and the effects of other GABArgic neurons. Is this related to issue #1?

3) It would be helpful to start the description of experiments in methods with the purpose/hypothesis of experiments.

4) In the methods, page 7, under the whole cell recording, the coordinates, and the procedure of AAV2/9-CaMKIIα-mCherry injection should be fully explained. What is the origin of the virus? How much virus injected and what is the titer?

5) All figures and legends are confusing and hard to follow. There are incorrect labeling and insufficient description of experiments in legends. The legend should have a full description of figures including full names of acronyms, name of the stain and color, etc.

Example:

- Figure 1 legend: SNI, EPM, OFT full name should be provided.

- In all figures, the numbers, titles, and graphs should be aligned.

- Figure legends lack detailed descriptions of the experiments, images, and graphs.

6) Figure 2: what is the condition of animals in the schematic and images of A, B, and C? The figure legend should be more detailed. Consistent presentations of significance (p values) of all graphs should be there, only can one p value is found for (I).

7) Figure 3 to 6: the same comment as in point#6 for Figure 2. There is inconsistency in the font all over the main text and in the figures.

8) Major for statistics: include confidence intervals, and replace all graphs showing comparisons between conditions (e.g. histograms) with Cumming estimation plots (https://www.estimationstats.com/#/)

9) Consider down tuning your comments as there is a tendency to overinterpret the results.

10) A rigorous grammar revision is needed (consider using web-based programs such as Grammarly).

## Author Response

Dear editor and referees, We are grateful to you for providing us an opportunity to revise our manuscript entitled "Modulation of Comorbid Chronic Neuropathic Pain and Anxiety-Like Behaviors by Glutamatergic Neurons in the vlPAG and the Analgesic and Anxiolytic Effects of EA" (Manuscript ID: eN-NWR-0454-23R1).

We are grateful to the constructive comments of the reviewing editor and reviewers. We believe your suggestions and comments have helped to improve the quality of the manuscript significantly. We have revised our manuscript based on the comments of the reviewers and provided point-by-point responses to all their concerns. The corresponding changes are highlighted in red.

We thank you for your suggestions on our manuscript and look forward to your favorable decision.

1. In some figure legends, the sample size (n) is presented as the number of slices from 3 mice. The same applied for the number of spiked neurons.

Response: Thank you for your valuable advice. We have revised the sample size (n) in the figure legends to n = 15 slices/group; N = 3 mice/group or n = 10 neurons/group; N = 3-4 mice/group (highlighted in red, page 13-14 Figure 2 legends, page 16-17 Figure 3 legends, page 22 Figure 5 legends).

2. The usage of 'n' and 'N' for sample sizes is inconsistent and confusing. For example, in figures 2 and 3C, 'n' is used to represent 15 slices from 3 mice, while on the graph of these data, 'N' is used instead of 'n.' In other graphs, 'N' represents the number of animals. This should be consistent throughout the manuscript. Assign N for animals all over the manuscript. Make n of other slices or neurons analyzed and make changes on the charts accordingly.

Response: Thank you for your kind suggestions. We have harmonized the use of 'n' and 'N' in the figures and legends. Use n to represent the number of slices or neurons in Figure 2-5. Assign N to represent the number of animals in Figure 1-6 (highlighted in red, page 13-14 Figure 2 legends, page 16-17 Figure 3 legends, page 22 Figure 5 legends).
